# Diisobutyl 4-(3-eth­oxy-4-hy­droxy­phen­yl)-2,6-dimethyl-1,4-dihydro­pyridine-3,5-dicarboxyl­ate

**DOI:** 10.1107/S1600536811055334

**Published:** 2012-01-07

**Authors:** Hoong-Kun Fun, Madhukar Hemamalini, B. Palakshi Reddy, V. Vijayakumar, S. Sarveswari

**Affiliations:** a X-ray Crystallography Unit, School of Physics, Universiti Sains Malaysia, 11800 USM, Penang, Malaysia; b Organic Chemistry Division, School of Advanced Sciences, VIT University, Vellore 632 014, India

## Abstract

The asymmetric unit of the title compound, C_25_H_35_NO_6_, contains two independent mol­ecules. In each mol­ecule, the 1,4-dihydro­pyridine ring adopts a flattened boat conformation. The dihedral angles between the 1,4-dihydro­pyridine and benzene rings are 87.55 (7) and 87.23 (7)°. In one of these mol­ecules, one of the isobutyl groups is disordered over two sets of sites, with an occupancy ratio of 0.890 (2):0.110 (2). In the crystal, mol­ecules are linked through N—H⋯O, O—H⋯O and C—H⋯O hydrogen bonds forming two-dimensional networks parallel to the *ab* plane. The crystal structure is further stabilized by weak C—H⋯π inter­actions.

## Related literature

For details and applications of dihydro­pyridines, see: Gaudio *et al.* (1994 ▶); Sunkel *et al.* (1992 ▶); Chapman *et al.* (1984 ▶); Peri *et al.* (2000 ▶); Zhou *et al.* (2005 ▶). For related structures, see: Palakshi Reddy *et al.* (2011*a* ▶,*b* ▶); Rathore *et al.* (2009 ▶). For reference bond-length data, see: Allen *et al.* (1987 ▶). For the stability of the temperature controller used in the data collection, see: Cosier & Glazer (1986 ▶). For ring comformations, see: Cremer & Pople (1975 ▶).
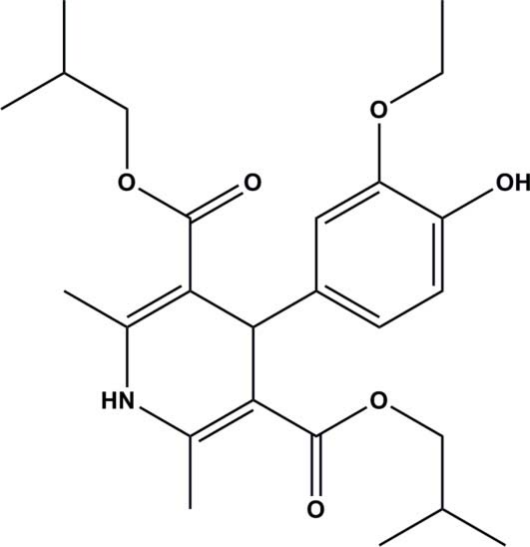


## Experimental

### 

#### Crystal data

C_25_H_35_NO_6_
*M* *_r_* = 445.54
Triclinic, 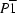

*a* = 12.7346 (2) Å
*b* = 13.1180 (2) Å
*c* = 15.7404 (2) Å
α = 71.766 (1)°
β = 89.813 (1)°
γ = 76.150 (1)°
*V* = 2417.50 (6) Å^3^
*Z* = 4
Mo *K*α radiation
μ = 0.09 mm^−1^
*T* = 100 K
0.59 × 0.23 × 0.13 mm

#### Data collection

Bruker SMART APEXII CCD area-detector diffractometer
Absorption correction: multi-scan (*SADABS*; Bruker, 2009 ▶) *T*_min_ = 0.951, *T*_max_ = 0.989
33902 measured reflections
14658 independent reflections
10852 reflections with *I* > 2σ(*I*)
*R*_int_ = 0.030

#### Refinement

*R*[*F* ^2^ > 2σ(*F* ^2^)] = 0.047
*wR*(*F* ^2^) = 0.125
*S* = 1.04
14658 reflections
619 parameters
3 restraints
H atoms treated by a mixture of independent and constrained refinement
Δρ_max_ = 0.43 e Å^−3^
Δρ_min_ = −0.25 e Å^−3^

### 

Data collection: *APEX2* (Bruker, 2009 ▶); cell refinement: *SAINT* (Bruker, 2009 ▶); data reduction: *SAINT*; program(s) used to solve structure: *SHELXTL* (Sheldrick, 2008 ▶); program(s) used to refine structure: *SHELXTL*; molecular graphics: *SHELXTL*; software used to prepare material for publication: *SHELXTL* and *PLATON* (Spek, 2009 ▶).

## Supplementary Material

Crystal structure: contains datablock(s) global, I. DOI: 10.1107/S1600536811055334/wn2463sup1.cif

Structure factors: contains datablock(s) I. DOI: 10.1107/S1600536811055334/wn2463Isup2.hkl

Supplementary material file. DOI: 10.1107/S1600536811055334/wn2463Isup3.cml

Additional supplementary materials:  crystallographic information; 3D view; checkCIF report

## Figures and Tables

**Table 1:** Hydrogen-bond geometry (Å, °) *Cg*2 and *Cg*4 are the centroids of the C18*A*–C23*A* and C18*B*–C23*B* rings, respectively.

*D*—H⋯*A*	*D*—H	H⋯*A*	*D*⋯*A*	*D*—H⋯*A*
N1*B*—H1*NB*⋯O6*A*^i^	0.896 (16)	2.077 (16)	2.9432 (14)	162.3 (15)
N1*A*—H1*NA*⋯O6*B*^ii^	0.893 (18)	2.067 (18)	2.9360 (14)	164.1 (16)
O6*B*—H1*OB*⋯O2*B*^ii^	0.83 (2)	1.98 (2)	2.7339 (13)	149.9 (19)
O6*A*—H1*OA*⋯O2*A*^iii^	0.83 (2)	1.94 (2)	2.6992 (13)	153 (2)
C11*A*—H11*C*⋯O4*B*^iv^	0.98	2.52	3.4755 (19)	164
C16*A*—H16*A*⋯O4*A*^v^	0.98	2.59	3.316 (2)	131
C24*A*—H24*B*⋯*Cg*2^iii^	0.99	2.99	3.7291 (15)	132
C24*B*—H24*C*⋯*Cg*4^ii^	0.99	2.94	3.6754 (14)	132
C14*X*—H14*C*⋯*Cg*4^vi^	1.00	2.98	3.978 (12)	172

Symmetry codes: (i) 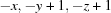; (ii) 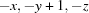; (iii) 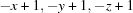; (iv) 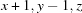; (v) 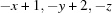; (vi) 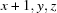.

**Fig. 1.**
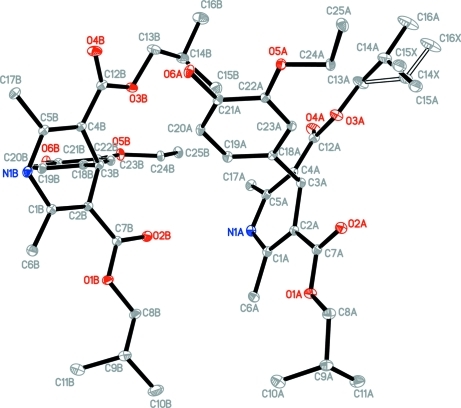
The asymmetric unit of the title compound, showing 50% probability displacement ellipsoids. H atoms have been omitted for clarity.

**Fig. 2.**
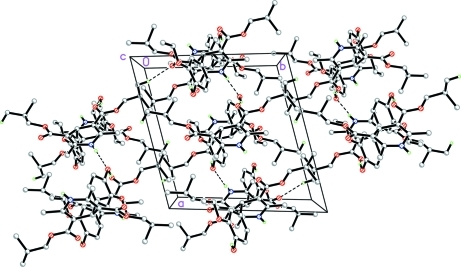
The crystal packing of title compound. Dashed lines indicate hydrogen bonds. Hydrogen atoms not involved in these hydrogen bonds have been omitted.

## supplementary crystallographic information

### Comment

1,4-Dihydropyridine (1,4-DHPs) derivatives are an important class of heterocycles owing to their potential biological activity and therapeutics uses such as antihypertensive (Gaudio *et al.*, 1994) and calcium channel modulators of the nifedipine type (Sunkel *et al.*, 1992). The presence of ester groups at the 3- and 5-positions on the 1,4-DHP ring is of crucial importance for its pharmacological effects. As a result, newly synthesized 1,4-DHPs possess different pharmacological activities, such as anticancer, bronchodilating (Chapman *et al.* 1984), antidiabetic, neurotropic, antianginal (Peri *et al.*, 2000) and other pharmacological activities (Zhou *et al.*, 2005). In continuation of our earlier interest in 1,4-DHPs (Palakshi Reddy *et al.*, 2011**a*,b*; Rathore *et al.*, 2009) we report here the synthesis and crystal structure of diisobutyl 4-(3-ethoxy-4-hydroxyphenyl)-2,6-dimethyl-1,4-dihydropyridine- 3,5-dicarboxylate.

The asymmetric unit of the title compound, consists of two crystallographically independent molecules, A and B (Fig. 1). The bond lengths (Allen *et al.*, 1987) and angles of molecules A and B agree with each other and are within normal ranges . In each molecule, the 1,4-dihydropyridine (N1A/C1A–C5A : N1B/C1B–C5B) ring adopts a flattened boat conformation with puckering parameters (Cremer & Pople, 1975) Q = 0.3036 (13) Å, θ = 73.2 (2) ° and φ = 184.3 (3)° for molecule A and Q = 0.3034 (13) Å, θ = 107. 5 (2)° and φ = 5.0 (3)° for molecule B. The dihedral angles between the two rings (C1A–C2A–C4A–C5A)/(C18A–C23A) and (C1B–C2B–C4B–C5B)/(C18B–C23B) are 87.55 (7)° and 87.23 (7)° respectively. In molecule A, one of the isobutyl groups is disordered over two positions with an occupancy ratio of 0.890 (2):0.110 (2).

In the crystal structure (Fig. 2), the molecules are linked through intermolecular N—H···O, O—H···O and C—H···O hydrogen bonds (Table 1) forming two-dimensional networks parallel to the *ab*-plane. The crystal structure is further stabilized by weak C—H···π interactions involving the centroids of the C18A–C23A (Cg2) and C18B–C23B (Cg4) rings.

### Experimental

A mixture of 4-hydroxy-3-ethoxybenzaldehyde (1 mmol), isobutyl acetoacetate (2 mmol) and ammonium acetate (1 mmol) were mixed along with 10 ml of ethanol and then refluxed for about 2 hours. The progress of the reaction was monitored by TLC. After confirming that the reaction was completed, the reaction mixture was cooled to room temperature and allowed to stand for 2 days to allow the formation of solid. The resulting solid product was washed with diethyl ether and recrystallized from ethanol to yield yellow crystals; M.p.: 140–142°C; Yield: 82%.

### Refinement

Atoms H1NA, H1NB, H1OA and H1OB were located in difference Fourier maps and refined freely [N—H = 0.894 (17)–0.896 (16) Å and O—H = 0.83 (2) Å]. The remaining H atoms were positioned geometrically and refined using a riding model, C—H = 0.95 Å for Csp^2^, 0.98 Å for methyl C, 0.99 Å for methyl C and 1.00 Å for methine C. *U*_iso_(H) = x*U*_eq_(C), where x = 1.5 for methyl H and 1.2 for all other H atoms.

### Crystal data

**Table:**

C_25_H_35_NO_6_	*Z* = 4
*M**_r_* = 445.54	*F*(000) = 960
Triclinic, *P*1	*D*_x_ = 1.224 Mg m^−^^3^
Hall symbol: -P 1	Mo *K*α radiation, λ = 0.71073 Å
*a* = 12.7346 (2) Å	Cell parameters from 9498 reflections
*b* = 13.1180 (2) Å	θ = 2.5–30.5°
*c* = 15.7404 (2) Å	µ = 0.09 mm^−^^1^
α = 71.766 (1)°	*T* = 100 K
β = 89.813 (1)°	Block, colourless
γ = 76.150 (1)°	0.59 × 0.23 × 0.13 mm
*V* = 2417.50 (6) Å^3^	

### Data collection

**Table:**

Bruker SMART APEXII CCD area-detector diffractometer	14658 independent reflections
Radiation source: fine-focus sealed tube	10852 reflections with *I* > 2σ(*I*)
graphite	*R*_int_ = 0.030
φ and ω scans	θ_max_ = 30.6°, θ_min_ = 1.7°
Absorption correction: multi-scan (*SADABS*; Bruker, 2009)	*h* = −18→18
*T*_min_ = 0.951, *T*_max_ = 0.989	*k* = −18→18
33902 measured reflections	*l* = −22→22

### Refinement

**Table:**

Refinement on *F*^2^	Primary atom site location: structure-invariant direct methods
Least-squares matrix: full	Secondary atom site location: difference Fourier map
*R*[*F*^2^ > 2σ(*F*^2^)] = 0.047	Hydrogen site location: inferred from neighbouring sites
*wR*(*F*^2^) = 0.125	H atoms treated by a mixture of independent and constrained refinement
*S* = 1.04	*w* = 1/[σ^2^(*F*_o_^2^) + (0.0575*P*)^2^ + 0.4282*P*] where *P* = (*F*_o_^2^ + 2*F*_c_^2^)/3
14658 reflections	(Δ/σ)_max_ < 0.001
619 parameters	Δρ_max_ = 0.43 e Å^−^^3^
3 restraints	Δρ_min_ = −0.25 e Å^−^^3^

### Special details

**Table:**

Experimental. The crystal was placed in the cold stream of an Oxford Cryosystems Cobra open-flow nitrogen cryostat (Cosier & Glazer, 1986) operating at 100.0 (1) K.
Geometry. All s.u.'s (except the s.u. in the dihedral angle between two l.s. planes) are estimated using the full covariance matrix. The cell s.u.'s are taken into account individually in the estimation of s.u.'s in distances, angles and torsion angles; correlations between s.u.'s in cell parameters are only used when they are defined by crystal symmetry. An approximate (isotropic) treatment of cell s.u.'s is used for estimating s.u.'s involving l.s. planes.
Refinement. Refinement of F^2^ against ALL reflections. The weighted R-factor wR and goodness of fit S are based on F^2^, conventional R-factors R are based on F, with F set to zero for negative F^2^. The threshold expression of F^2^ > 2σ(F^2^) is used only for calculating R-factors(gt) etc. and is not relevant to the choice of reflections for refinement. R-factors based on F^2^ are statistically about twice as large as those based on F, and R- factors based on ALL data will be even larger.

### Fractional atomic coordinates and isotropic or equivalent isotropic displacement parameters (Å^2^)

**Table:**

	*x*	*y*	*z*	*U*_iso_*/*U*_eq_	Occ. (<1)
O1A	0.65593 (7)	0.22427 (7)	0.32513 (6)	0.01877 (18)	
O2A	0.68239 (7)	0.37473 (7)	0.35316 (6)	0.01794 (17)	
O3A	0.54155 (7)	0.73836 (7)	0.14127 (6)	0.02162 (19)	
O4A	0.46664 (9)	0.75911 (8)	0.00632 (6)	0.0301 (2)	
O5A	0.45070 (6)	0.68825 (7)	0.47222 (6)	0.01704 (17)	
O6A	0.24903 (7)	0.65738 (8)	0.47620 (6)	0.01842 (18)	
N1A	0.41808 (8)	0.43615 (9)	0.12188 (7)	0.0168 (2)	
C1A	0.49199 (9)	0.36210 (10)	0.19006 (8)	0.0154 (2)	
C2A	0.55112 (9)	0.40291 (10)	0.23764 (8)	0.0139 (2)	
C3A	0.52654 (9)	0.52684 (9)	0.22317 (8)	0.0138 (2)	
H3AA	0.5968	0.5458	0.2304	0.017*	
C4A	0.47737 (9)	0.59128 (10)	0.12786 (8)	0.0148 (2)	
C5A	0.41768 (9)	0.54670 (10)	0.08484 (8)	0.0158 (2)	
C6A	0.49800 (10)	0.24288 (10)	0.20069 (9)	0.0191 (2)	
H6AA	0.4892	0.2033	0.2633	0.029*	
H6AB	0.5686	0.2085	0.1839	0.029*	
H6AC	0.4401	0.2391	0.1618	0.029*	
C7A	0.63478 (9)	0.33471 (10)	0.30929 (8)	0.0148 (2)	
C8A	0.72798 (10)	0.15556 (10)	0.40420 (9)	0.0207 (2)	
H8AA	0.6908	0.1582	0.4591	0.025*	
H8AB	0.7935	0.1835	0.4047	0.025*	
C9A	0.75998 (11)	0.03710 (11)	0.40238 (9)	0.0216 (3)	
H9AA	0.8038	−0.0091	0.4600	0.026*	
C10A	0.66240 (12)	−0.01007 (12)	0.39875 (11)	0.0296 (3)	
H10A	0.6164	0.0000	0.4470	0.044*	
H10B	0.6874	−0.0893	0.4062	0.044*	
H10C	0.6206	0.0288	0.3407	0.044*	
C11A	0.83235 (12)	0.02594 (12)	0.32709 (10)	0.0295 (3)	
H11A	0.8961	0.0535	0.3329	0.044*	
H11B	0.7919	0.0693	0.2691	0.044*	
H11C	0.8557	−0.0522	0.3307	0.044*	
C12A	0.49199 (10)	0.70315 (10)	0.08419 (8)	0.0180 (2)	
C13A	0.56687 (13)	0.84422 (11)	0.10141 (10)	0.0301 (3)	
H13A	0.4996	0.9022	0.0746	0.036*	0.890 (2)
H13B	0.6165	0.8401	0.0534	0.036*	0.890 (2)
H13E	0.5121	0.9018	0.1129	0.036*	0.110 (2)
H13F	0.5675	0.8600	0.0376	0.036*	0.110 (2)
C14A	0.62011 (13)	0.87297 (12)	0.17352 (11)	0.0249 (3)	0.890 (2)
H14A	0.5686	0.8770	0.2211	0.030*	0.890 (2)
C15A	0.72407 (14)	0.78674 (14)	0.21621 (13)	0.0318 (4)	0.890 (2)
H15A	0.7080	0.7146	0.2438	0.048*	0.890 (2)
H15B	0.7562	0.8083	0.2623	0.048*	0.890 (2)
H15C	0.7752	0.7815	0.1702	0.048*	0.890 (2)
C16A	0.6415 (2)	0.98703 (15)	0.13148 (15)	0.0487 (6)	0.890 (2)
H16A	0.5728	1.0419	0.1069	0.073*	0.890 (2)
H16B	0.6900	0.9850	0.0832	0.073*	0.890 (2)
H16C	0.6756	1.0075	0.1773	0.073*	0.890 (2)
C14X	0.6819 (10)	0.8431 (9)	0.1393 (8)	0.0249 (3)	0.110 (2)
H14C	0.7387	0.7814	0.1294	0.030*	0.110 (2)
C15X	0.6729 (12)	0.8237 (12)	0.2361 (9)	0.0318 (4)	0.110 (2)
H15G	0.7407	0.8266	0.2634	0.048*	0.110 (2)
H15H	0.6591	0.7506	0.2642	0.048*	0.110 (2)
H15I	0.6129	0.8810	0.2451	0.048*	0.110 (2)
C16X	0.7096 (17)	0.9539 (12)	0.0949 (11)	0.0487 (6)	0.110 (2)
H16G	0.7460	0.9740	0.1398	0.073*	0.110 (2)
H16H	0.6428	1.0114	0.0692	0.073*	0.110 (2)
H16I	0.7578	0.9474	0.0472	0.073*	0.110 (2)
C17A	0.34730 (10)	0.60315 (11)	−0.00102 (8)	0.0194 (2)	
H17A	0.3303	0.6836	−0.0137	0.029*	
H17B	0.2799	0.5788	0.0046	0.029*	
H17C	0.3857	0.5840	−0.0502	0.029*	
C18A	0.45270 (9)	0.55975 (9)	0.29224 (8)	0.0138 (2)	
C19A	0.34741 (9)	0.54636 (10)	0.29630 (8)	0.0164 (2)	
H19A	0.3211	0.5147	0.2567	0.020*	
C20A	0.28027 (9)	0.57908 (10)	0.35819 (8)	0.0161 (2)	
H20A	0.2085	0.5697	0.3604	0.019*	
C21A	0.31732 (9)	0.62515 (9)	0.41644 (8)	0.0145 (2)	
C22A	0.42302 (9)	0.63953 (10)	0.41269 (8)	0.0143 (2)	
C23A	0.48984 (9)	0.60620 (10)	0.35129 (8)	0.0147 (2)	
H23A	0.5618	0.6151	0.3494	0.018*	
C24A	0.56215 (9)	0.69126 (11)	0.47879 (9)	0.0180 (2)	
H24A	0.5817	0.7362	0.4206	0.022*	
H24B	0.6098	0.6153	0.4947	0.022*	
C25A	0.57646 (11)	0.74199 (12)	0.55053 (9)	0.0226 (3)	
H25A	0.6520	0.7456	0.5558	0.034*	
H25B	0.5580	0.6963	0.6080	0.034*	
H25C	0.5286	0.8170	0.5343	0.034*	
O1B	0.16199 (7)	0.21473 (7)	0.32189 (6)	0.01890 (18)	
O2B	0.18733 (7)	0.36622 (7)	0.21617 (6)	0.01867 (18)	
O3B	0.03283 (7)	0.73069 (7)	0.23134 (6)	0.01937 (18)	
O4B	−0.03557 (8)	0.75077 (8)	0.35879 (6)	0.0256 (2)	
O5B	−0.05533 (7)	0.68711 (7)	−0.06563 (6)	0.01869 (18)	
O6B	−0.25198 (7)	0.64538 (8)	−0.05293 (6)	0.01979 (18)	
N1B	−0.07913 (8)	0.42474 (9)	0.41615 (7)	0.0163 (2)	
C1B	−0.00455 (9)	0.35099 (10)	0.38633 (8)	0.0151 (2)	
C2B	0.05339 (9)	0.39218 (9)	0.31639 (8)	0.0141 (2)	
C3B	0.02680 (9)	0.51597 (9)	0.26542 (8)	0.0137 (2)	
H3BA	0.0965	0.5360	0.2473	0.016*	
C4B	−0.02275 (9)	0.58084 (10)	0.32699 (8)	0.0148 (2)	
C5B	−0.08165 (9)	0.53597 (10)	0.39405 (8)	0.0154 (2)	
C6B	0.00315 (10)	0.23158 (10)	0.43862 (8)	0.0192 (2)	
H6BA	−0.0067	0.1916	0.3972	0.029*	
H6BB	−0.0534	0.2274	0.4810	0.029*	
H6BC	0.0746	0.1979	0.4717	0.029*	
C7B	0.13895 (9)	0.32552 (10)	0.28006 (8)	0.0151 (2)	
C8B	0.24295 (10)	0.14910 (10)	0.28228 (9)	0.0217 (3)	
H8BA	0.3091	0.1772	0.2756	0.026*	
H8BB	0.2147	0.1547	0.2220	0.026*	
C9B	0.27000 (10)	0.02944 (11)	0.34241 (9)	0.0221 (3)	
H9BA	0.2986	0.0260	0.4026	0.027*	
C10B	0.35969 (13)	−0.03770 (12)	0.30230 (12)	0.0351 (3)	
H10D	0.4231	−0.0067	0.2970	0.053*	
H10E	0.3334	−0.0343	0.2428	0.053*	
H10F	0.3799	−0.1151	0.3414	0.053*	
C11B	0.17072 (12)	−0.01796 (11)	0.35487 (10)	0.0271 (3)	
H11D	0.1181	0.0216	0.3865	0.041*	
H11E	0.1926	−0.0969	0.3901	0.041*	
H11F	0.1376	−0.0091	0.2961	0.041*	
C12B	−0.01152 (9)	0.69405 (10)	0.30992 (8)	0.0170 (2)	
C13B	0.05064 (12)	0.84019 (11)	0.21072 (9)	0.0262 (3)	
H13C	0.1020	0.8415	0.2572	0.031*	
H13D	−0.0187	0.8947	0.2095	0.031*	
C14B	0.09665 (11)	0.86980 (11)	0.12014 (9)	0.0239 (3)	
H14B	0.0466	0.8607	0.0758	0.029*	
C15B	0.20785 (13)	0.79489 (13)	0.12074 (12)	0.0364 (4)	
H15D	0.2027	0.7179	0.1369	0.055*	
H15E	0.2580	0.8015	0.1647	0.055*	
H15F	0.2347	0.8169	0.0610	0.055*	
C16B	0.10039 (14)	0.99152 (12)	0.09083 (11)	0.0345 (3)	
H16D	0.0274	1.0383	0.0897	0.052*	
H16E	0.1266	1.0120	0.0307	0.052*	
H16F	0.1495	1.0023	0.1333	0.052*	
C17B	−0.15385 (10)	0.59335 (11)	0.44969 (8)	0.0186 (2)	
H17D	−0.1756	0.6732	0.4177	0.028*	
H17E	−0.1145	0.5798	0.5071	0.028*	
H17F	−0.2186	0.5644	0.4606	0.028*	
C18B	−0.04746 (9)	0.54802 (9)	0.18026 (8)	0.0142 (2)	
C19B	−0.15115 (9)	0.53000 (10)	0.18359 (8)	0.0162 (2)	
H19B	−0.1759	0.4950	0.2397	0.019*	
C20B	−0.21870 (9)	0.56281 (10)	0.10547 (8)	0.0164 (2)	
H20B	−0.2892	0.5500	0.1086	0.020*	
C21B	−0.18408 (9)	0.61395 (10)	0.02332 (8)	0.0152 (2)	
C22B	−0.08020 (9)	0.63357 (10)	0.01904 (8)	0.0151 (2)	
C23B	−0.01288 (9)	0.59957 (10)	0.09687 (8)	0.0150 (2)	
H23B	0.0580	0.6114	0.0936	0.018*	
C24B	0.05475 (9)	0.69473 (10)	−0.07449 (8)	0.0177 (2)	
H24C	0.1047	0.6197	−0.0532	0.021*	
H24D	0.0724	0.7385	−0.0380	0.021*	
C25B	0.06735 (11)	0.75040 (11)	−0.17204 (9)	0.0225 (3)	
H25D	0.1421	0.7570	−0.1795	0.034*	
H25E	0.0174	0.8244	−0.1926	0.034*	
H25F	0.0507	0.7060	−0.2075	0.034*	
H1NB	−0.1208 (12)	0.3973 (13)	0.4590 (11)	0.025 (4)*	
H1NA	0.3762 (13)	0.4104 (14)	0.0925 (12)	0.031 (4)*	
H1OB	−0.2134 (15)	0.6488 (16)	−0.0958 (13)	0.044 (5)*	
H1OA	0.2866 (16)	0.6559 (17)	0.5198 (15)	0.052 (6)*	

### Atomic displacement parameters (Å^2^)

**Table:**

	*U*^11^	*U*^22^	*U*^33^	*U*^12^	*U*^13^	*U*^23^
O1A	0.0212 (4)	0.0158 (4)	0.0176 (4)	−0.0015 (3)	−0.0053 (3)	−0.0052 (3)
O2A	0.0202 (4)	0.0195 (4)	0.0148 (4)	−0.0053 (3)	−0.0024 (3)	−0.0060 (3)
O3A	0.0303 (5)	0.0175 (4)	0.0165 (4)	−0.0079 (4)	−0.0046 (4)	−0.0032 (3)
O4A	0.0471 (6)	0.0257 (5)	0.0152 (5)	−0.0134 (4)	−0.0056 (4)	0.0000 (4)
O5A	0.0146 (4)	0.0236 (4)	0.0181 (4)	−0.0066 (3)	0.0030 (3)	−0.0126 (4)
O6A	0.0142 (4)	0.0293 (5)	0.0146 (4)	−0.0049 (3)	0.0029 (3)	−0.0115 (4)
N1A	0.0162 (4)	0.0217 (5)	0.0134 (5)	−0.0057 (4)	−0.0015 (4)	−0.0062 (4)
C1A	0.0152 (5)	0.0187 (6)	0.0123 (5)	−0.0044 (4)	0.0019 (4)	−0.0048 (4)
C2A	0.0139 (5)	0.0169 (5)	0.0111 (5)	−0.0035 (4)	0.0019 (4)	−0.0052 (4)
C3A	0.0143 (5)	0.0166 (5)	0.0112 (5)	−0.0041 (4)	0.0015 (4)	−0.0054 (4)
C4A	0.0145 (5)	0.0177 (6)	0.0105 (5)	−0.0018 (4)	0.0004 (4)	−0.0039 (4)
C5A	0.0136 (5)	0.0212 (6)	0.0118 (5)	−0.0021 (4)	0.0016 (4)	−0.0058 (4)
C6A	0.0219 (6)	0.0199 (6)	0.0174 (6)	−0.0070 (5)	−0.0011 (5)	−0.0072 (5)
C7A	0.0145 (5)	0.0172 (5)	0.0127 (5)	−0.0033 (4)	0.0020 (4)	−0.0056 (4)
C8A	0.0224 (6)	0.0184 (6)	0.0180 (6)	−0.0006 (5)	−0.0051 (5)	−0.0046 (5)
C9A	0.0257 (6)	0.0179 (6)	0.0194 (6)	−0.0031 (5)	−0.0003 (5)	−0.0050 (5)
C10A	0.0348 (7)	0.0236 (7)	0.0304 (8)	−0.0109 (6)	0.0025 (6)	−0.0060 (6)
C11A	0.0342 (7)	0.0230 (7)	0.0301 (8)	−0.0040 (6)	0.0090 (6)	−0.0092 (6)
C12A	0.0191 (5)	0.0196 (6)	0.0148 (6)	−0.0030 (4)	0.0002 (4)	−0.0063 (5)
C13A	0.0453 (8)	0.0194 (6)	0.0233 (7)	−0.0120 (6)	−0.0109 (6)	−0.0005 (5)
C14A	0.0321 (8)	0.0182 (7)	0.0234 (8)	−0.0038 (6)	−0.0047 (6)	−0.0070 (6)
C15A	0.0289 (8)	0.0236 (8)	0.0414 (10)	−0.0027 (6)	−0.0101 (7)	−0.0112 (7)
C16A	0.0825 (16)	0.0215 (9)	0.0408 (12)	−0.0187 (10)	−0.0254 (11)	−0.0037 (8)
C14X	0.0321 (8)	0.0182 (7)	0.0234 (8)	−0.0038 (6)	−0.0047 (6)	−0.0070 (6)
C15X	0.0289 (8)	0.0236 (8)	0.0414 (10)	−0.0027 (6)	−0.0101 (7)	−0.0112 (7)
C16X	0.0825 (16)	0.0215 (9)	0.0408 (12)	−0.0187 (10)	−0.0254 (11)	−0.0037 (8)
C17A	0.0179 (5)	0.0245 (6)	0.0140 (6)	−0.0022 (5)	−0.0025 (4)	−0.0062 (5)
C18A	0.0140 (5)	0.0147 (5)	0.0113 (5)	−0.0018 (4)	0.0009 (4)	−0.0037 (4)
C19A	0.0164 (5)	0.0210 (6)	0.0131 (5)	−0.0052 (4)	−0.0004 (4)	−0.0068 (4)
C20A	0.0127 (5)	0.0214 (6)	0.0148 (5)	−0.0052 (4)	0.0009 (4)	−0.0058 (4)
C21A	0.0140 (5)	0.0161 (5)	0.0113 (5)	−0.0012 (4)	0.0018 (4)	−0.0036 (4)
C22A	0.0152 (5)	0.0157 (5)	0.0129 (5)	−0.0036 (4)	0.0004 (4)	−0.0058 (4)
C23A	0.0136 (5)	0.0164 (5)	0.0149 (5)	−0.0040 (4)	0.0015 (4)	−0.0059 (4)
C24A	0.0145 (5)	0.0230 (6)	0.0196 (6)	−0.0070 (4)	0.0023 (4)	−0.0094 (5)
C25A	0.0234 (6)	0.0299 (7)	0.0207 (6)	−0.0123 (5)	0.0035 (5)	−0.0125 (5)
O1B	0.0211 (4)	0.0148 (4)	0.0198 (4)	−0.0023 (3)	0.0066 (3)	−0.0058 (3)
O2B	0.0206 (4)	0.0200 (4)	0.0162 (4)	−0.0049 (3)	0.0052 (3)	−0.0070 (3)
O3B	0.0268 (4)	0.0165 (4)	0.0170 (4)	−0.0073 (3)	0.0041 (4)	−0.0072 (3)
O4B	0.0371 (5)	0.0219 (5)	0.0219 (5)	−0.0071 (4)	0.0056 (4)	−0.0129 (4)
O5B	0.0162 (4)	0.0264 (5)	0.0118 (4)	−0.0080 (3)	0.0006 (3)	−0.0018 (3)
O6B	0.0156 (4)	0.0326 (5)	0.0115 (4)	−0.0072 (4)	−0.0006 (3)	−0.0067 (4)
N1B	0.0156 (4)	0.0197 (5)	0.0146 (5)	−0.0048 (4)	0.0044 (4)	−0.0069 (4)
C1B	0.0149 (5)	0.0180 (5)	0.0132 (5)	−0.0035 (4)	0.0001 (4)	−0.0065 (4)
C2B	0.0143 (5)	0.0156 (5)	0.0129 (5)	−0.0030 (4)	0.0007 (4)	−0.0058 (4)
C3B	0.0138 (5)	0.0156 (5)	0.0121 (5)	−0.0030 (4)	0.0008 (4)	−0.0054 (4)
C4B	0.0144 (5)	0.0167 (5)	0.0129 (5)	−0.0011 (4)	−0.0009 (4)	−0.0065 (4)
C5B	0.0140 (5)	0.0188 (6)	0.0130 (5)	−0.0011 (4)	−0.0013 (4)	−0.0069 (4)
C6B	0.0229 (6)	0.0192 (6)	0.0164 (6)	−0.0068 (5)	0.0047 (5)	−0.0057 (5)
C7B	0.0152 (5)	0.0176 (5)	0.0138 (5)	−0.0039 (4)	0.0001 (4)	−0.0068 (4)
C8B	0.0224 (6)	0.0186 (6)	0.0231 (6)	−0.0013 (5)	0.0075 (5)	−0.0082 (5)
C9B	0.0249 (6)	0.0182 (6)	0.0210 (6)	−0.0014 (5)	0.0005 (5)	−0.0061 (5)
C10B	0.0350 (8)	0.0225 (7)	0.0414 (9)	0.0036 (6)	0.0068 (7)	−0.0095 (6)
C11B	0.0346 (7)	0.0202 (6)	0.0261 (7)	−0.0080 (5)	−0.0008 (6)	−0.0062 (5)
C12B	0.0173 (5)	0.0185 (6)	0.0142 (5)	−0.0015 (4)	−0.0008 (4)	−0.0063 (4)
C13B	0.0417 (8)	0.0175 (6)	0.0234 (7)	−0.0116 (6)	0.0083 (6)	−0.0091 (5)
C14B	0.0322 (7)	0.0195 (6)	0.0196 (6)	−0.0070 (5)	0.0028 (5)	−0.0053 (5)
C15B	0.0348 (8)	0.0304 (8)	0.0397 (9)	−0.0075 (6)	0.0124 (7)	−0.0060 (7)
C16B	0.0529 (9)	0.0219 (7)	0.0263 (8)	−0.0110 (7)	0.0048 (7)	−0.0034 (6)
C17B	0.0165 (5)	0.0225 (6)	0.0160 (6)	−0.0008 (4)	0.0019 (4)	−0.0081 (5)
C18B	0.0155 (5)	0.0142 (5)	0.0129 (5)	−0.0022 (4)	0.0003 (4)	−0.0054 (4)
C19B	0.0166 (5)	0.0198 (6)	0.0137 (5)	−0.0048 (4)	0.0030 (4)	−0.0075 (4)
C20B	0.0138 (5)	0.0212 (6)	0.0162 (6)	−0.0047 (4)	0.0023 (4)	−0.0085 (5)
C21B	0.0148 (5)	0.0190 (6)	0.0125 (5)	−0.0030 (4)	−0.0007 (4)	−0.0071 (4)
C22B	0.0166 (5)	0.0171 (5)	0.0120 (5)	−0.0047 (4)	0.0024 (4)	−0.0048 (4)
C23B	0.0137 (5)	0.0180 (5)	0.0141 (5)	−0.0047 (4)	0.0008 (4)	−0.0055 (4)
C24B	0.0163 (5)	0.0216 (6)	0.0156 (6)	−0.0075 (4)	0.0020 (4)	−0.0047 (5)
C25B	0.0243 (6)	0.0268 (7)	0.0168 (6)	−0.0106 (5)	0.0045 (5)	−0.0043 (5)

### Geometric parameters (Å, °)

**Table:**

O1A—C7A	1.3496 (14)	C24A—C25A	1.5103 (17)
O1A—C8A	1.4535 (15)	C24A—H24A	0.9900
O2A—C7A	1.2255 (14)	C24A—H24B	0.9900
O3A—C12A	1.3498 (15)	C25A—H25A	0.9800
O3A—C13A	1.4470 (16)	C25A—H25B	0.9800
O4A—C12A	1.2130 (15)	C25A—H25C	0.9800
O5A—C22A	1.3768 (14)	O1B—C7B	1.3528 (14)
O5A—C24A	1.4342 (13)	O1B—C8B	1.4490 (14)
O6A—C21A	1.3739 (13)	O2B—C7B	1.2220 (14)
O6A—H1OA	0.83 (2)	O3B—C12B	1.3540 (14)
N1A—C1A	1.3775 (15)	O3B—C13B	1.4436 (15)
N1A—C5A	1.3823 (16)	O4B—C12B	1.2192 (14)
N1A—H1NA	0.894 (17)	O5B—C22B	1.3739 (14)
C1A—C2A	1.3646 (16)	O5B—C24B	1.4324 (14)
C1A—C6A	1.5028 (17)	O6B—C21B	1.3728 (14)
C2A—C7A	1.4587 (16)	O6B—H1OB	0.83 (2)
C2A—C3A	1.5224 (16)	N1B—C1B	1.3782 (15)
C3A—C4A	1.5219 (16)	N1B—C5B	1.3824 (16)
C3A—C18A	1.5313 (15)	N1B—H1NB	0.896 (16)
C3A—H3AA	1.0000	C1B—C2B	1.3644 (16)
C4A—C5A	1.3573 (16)	C1B—C6B	1.5036 (17)
C4A—C12A	1.4695 (17)	C2B—C7B	1.4600 (16)
C5A—C17A	1.4974 (16)	C2B—C3B	1.5227 (16)
C6A—H6AA	0.9800	C3B—C4B	1.5221 (16)
C6A—H6AB	0.9800	C3B—C18B	1.5275 (16)
C6A—H6AC	0.9800	C3B—H3BA	1.0000
C8A—C9A	1.5187 (18)	C4B—C5B	1.3567 (16)
C8A—H8AA	0.9900	C4B—C12B	1.4663 (17)
C8A—H8AB	0.9900	C5B—C17B	1.5016 (16)
C9A—C11A	1.5205 (19)	C6B—H6BA	0.9800
C9A—C10A	1.5234 (19)	C6B—H6BB	0.9800
C9A—H9AA	1.0000	C6B—H6BC	0.9800
C10A—H10A	0.9800	C8B—C9B	1.5140 (18)
C10A—H10B	0.9800	C8B—H8BA	0.9900
C10A—H10C	0.9800	C8B—H8BB	0.9900
C11A—H11A	0.9800	C9B—C11B	1.5237 (19)
C11A—H11B	0.9800	C9B—C10B	1.5300 (19)
C11A—H11C	0.9800	C9B—H9BA	1.0000
C13A—C14A	1.512 (2)	C10B—H10D	0.9800
C13A—C14X	1.578 (12)	C10B—H10E	0.9800
C13A—H13A	0.9900	C10B—H10F	0.9800
C13A—H13B	0.9900	C11B—H11D	0.9800
C13A—H13E	0.9599	C11B—H11E	0.9800
C13A—H13F	0.9601	C11B—H11F	0.9800
C14A—C15A	1.516 (2)	C13B—C14B	1.5112 (19)
C14A—C16A	1.525 (2)	C13B—H13C	0.9900
C14A—H13E	1.5742	C13B—H13D	0.9900
C14A—H14A	1.0000	C14B—C15B	1.516 (2)
C15A—H15A	0.9800	C14B—C16B	1.5297 (19)
C15A—H15B	0.9800	C14B—H14B	1.0000
C15A—H15C	0.9800	C15B—H15D	0.9800
C16A—H16A	0.9800	C15B—H15E	0.9800
C16A—H16B	0.9800	C15B—H15F	0.9800
C16A—H16C	0.9800	C16B—H16D	0.9800
C14X—C15X	1.473 (14)	C16B—H16E	0.9800
C14X—C16X	1.528 (14)	C16B—H16F	0.9800
C14X—H14C	1.0000	C17B—H17D	0.9800
C15X—H15G	0.9800	C17B—H17E	0.9800
C15X—H15H	0.9800	C17B—H17F	0.9800
C15X—H15I	0.9800	C18B—C19B	1.3940 (16)
C16X—H16G	0.9800	C18B—C23B	1.3992 (16)
C16X—H16H	0.9800	C19B—C20B	1.3913 (17)
C16X—H16I	0.9800	C19B—H19B	0.9500
C17A—H17A	0.9800	C20B—C21B	1.3826 (16)
C17A—H17B	0.9800	C20B—H20B	0.9500
C17A—H17C	0.9800	C21B—C22B	1.4044 (15)
C18A—C19A	1.3921 (15)	C22B—C23B	1.3844 (16)
C18A—C23A	1.3979 (16)	C23B—H23B	0.9500
C19A—C20A	1.3947 (16)	C24B—C25B	1.5064 (17)
C19A—H19A	0.9500	C24B—H24C	0.9900
C20A—C21A	1.3837 (16)	C24B—H24D	0.9900
C20A—H20A	0.9500	C25B—H25D	0.9800
C21A—C22A	1.4018 (15)	C25B—H25E	0.9800
C22A—C23A	1.3893 (16)	C25B—H25F	0.9800
C23A—H23A	0.9500		
			
C7A—O1A—C8A	115.81 (9)	O5A—C24A—C25A	108.02 (10)
C12A—O3A—C13A	115.02 (10)	O5A—C24A—H24A	110.1
C22A—O5A—C24A	116.45 (9)	C25A—C24A—H24A	110.1
C21A—O6A—H1OA	108.3 (14)	O5A—C24A—H24B	110.1
C1A—N1A—C5A	123.43 (10)	C25A—C24A—H24B	110.1
C1A—N1A—H1NA	119.1 (11)	H24A—C24A—H24B	108.4
C5A—N1A—H1NA	116.5 (11)	C24A—C25A—H25A	109.5
C2A—C1A—N1A	118.43 (11)	C24A—C25A—H25B	109.5
C2A—C1A—C6A	128.06 (11)	H25A—C25A—H25B	109.5
N1A—C1A—C6A	113.50 (10)	C24A—C25A—H25C	109.5
C1A—C2A—C7A	124.52 (11)	H25A—C25A—H25C	109.5
C1A—C2A—C3A	120.63 (10)	H25B—C25A—H25C	109.5
C7A—C2A—C3A	114.69 (10)	C7B—O1B—C8B	115.11 (9)
C4A—C3A—C2A	109.76 (9)	C12B—O3B—C13B	115.26 (9)
C4A—C3A—C18A	111.26 (9)	C22B—O5B—C24B	116.07 (9)
C2A—C3A—C18A	111.97 (9)	C21B—O6B—H1OB	107.3 (13)
C4A—C3A—H3AA	107.9	C1B—N1B—C5B	123.42 (10)
C2A—C3A—H3AA	107.9	C1B—N1B—H1NB	118.0 (10)
C18A—C3A—H3AA	107.9	C5B—N1B—H1NB	117.8 (10)
C5A—C4A—C12A	120.46 (11)	C2B—C1B—N1B	118.46 (11)
C5A—C4A—C3A	119.97 (10)	C2B—C1B—C6B	128.02 (11)
C12A—C4A—C3A	119.54 (10)	N1B—C1B—C6B	113.51 (10)
C4A—C5A—N1A	119.07 (11)	C1B—C2B—C7B	125.15 (11)
C4A—C5A—C17A	127.95 (11)	C1B—C2B—C3B	120.62 (10)
N1A—C5A—C17A	112.97 (10)	C7B—C2B—C3B	114.13 (10)
C1A—C6A—H6AA	109.5	C4B—C3B—C2B	109.75 (9)
C1A—C6A—H6AB	109.5	C4B—C3B—C18B	110.92 (9)
H6AA—C6A—H6AB	109.5	C2B—C3B—C18B	112.61 (9)
C1A—C6A—H6AC	109.5	C4B—C3B—H3BA	107.8
H6AA—C6A—H6AC	109.5	C2B—C3B—H3BA	107.8
H6AB—C6A—H6AC	109.5	C18B—C3B—H3BA	107.8
O2A—C7A—O1A	121.84 (10)	C5B—C4B—C12B	120.54 (10)
O2A—C7A—C2A	122.32 (11)	C5B—C4B—C3B	119.91 (10)
O1A—C7A—C2A	115.83 (10)	C12B—C4B—C3B	119.46 (10)
O1A—C8A—C9A	108.88 (10)	C4B—C5B—N1B	119.12 (10)
O1A—C8A—H8AA	109.9	C4B—C5B—C17B	127.75 (11)
C9A—C8A—H8AA	109.9	N1B—C5B—C17B	113.12 (10)
O1A—C8A—H8AB	109.9	C1B—C6B—H6BA	109.5
C9A—C8A—H8AB	109.9	C1B—C6B—H6BB	109.5
H8AA—C8A—H8AB	108.3	H6BA—C6B—H6BB	109.5
C8A—C9A—C11A	111.75 (11)	C1B—C6B—H6BC	109.5
C8A—C9A—C10A	112.68 (11)	H6BA—C6B—H6BC	109.5
C11A—C9A—C10A	111.35 (12)	H6BB—C6B—H6BC	109.5
C8A—C9A—H9AA	106.9	O2B—C7B—O1B	121.61 (10)
C11A—C9A—H9AA	106.9	O2B—C7B—C2B	122.73 (11)
C10A—C9A—H9AA	106.9	O1B—C7B—C2B	115.66 (10)
C9A—C10A—H10A	109.5	O1B—C8B—C9B	108.93 (10)
C9A—C10A—H10B	109.5	O1B—C8B—H8BA	109.9
H10A—C10A—H10B	109.5	C9B—C8B—H8BA	109.9
C9A—C10A—H10C	109.5	O1B—C8B—H8BB	109.9
H10A—C10A—H10C	109.5	C9B—C8B—H8BB	109.9
H10B—C10A—H10C	109.5	H8BA—C8B—H8BB	108.3
C9A—C11A—H11A	109.5	C8B—C9B—C11B	112.04 (11)
C9A—C11A—H11B	109.5	C8B—C9B—C10B	108.33 (11)
H11A—C11A—H11B	109.5	C11B—C9B—C10B	111.42 (12)
C9A—C11A—H11C	109.5	C8B—C9B—H9BA	108.3
H11A—C11A—H11C	109.5	C11B—C9B—H9BA	108.3
H11B—C11A—H11C	109.5	C10B—C9B—H9BA	108.3
O4A—C12A—O3A	121.89 (11)	C9B—C10B—H10D	109.5
O4A—C12A—C4A	126.42 (11)	C9B—C10B—H10E	109.5
O3A—C12A—C4A	111.67 (10)	H10D—C10B—H10E	109.5
O3A—C13A—C14A	108.76 (11)	C9B—C10B—H10F	109.5
O3A—C13A—C14X	111.1 (4)	H10D—C10B—H10F	109.5
C14A—C13A—C14X	38.5 (4)	H10E—C10B—H10F	109.5
O3A—C13A—H13A	109.9	C9B—C11B—H11D	109.5
C14A—C13A—H13A	109.9	C9B—C11B—H11E	109.5
C14X—C13A—H13A	135.1	H11D—C11B—H11E	109.5
O3A—C13A—H13B	109.9	C9B—C11B—H11F	109.5
C14A—C13A—H13B	109.9	H11D—C11B—H11F	109.5
C14X—C13A—H13B	73.5	H11E—C11B—H11F	109.5
H13A—C13A—H13B	108.3	O4B—C12B—O3B	121.85 (11)
O3A—C13A—H13E	109.7	O4B—C12B—C4B	126.63 (11)
C14A—C13A—H13E	75.5	O3B—C12B—C4B	111.51 (10)
C14X—C13A—H13E	110.0	O3B—C13B—C14B	108.56 (10)
H13A—C13A—H13E	37.2	O3B—C13B—H13C	110.0
H13B—C13A—H13E	135.2	C14B—C13B—H13C	110.0
O3A—C13A—H13F	109.4	O3B—C13B—H13D	110.0
C14A—C13A—H13F	137.4	C14B—C13B—H13D	110.0
C14X—C13A—H13F	108.4	H13C—C13B—H13D	108.4
H13A—C13A—H13F	73.5	C13B—C14B—C15B	112.08 (12)
H13B—C13A—H13F	37.8	C13B—C14B—C16B	109.32 (11)
H13E—C13A—H13F	108.1	C15B—C14B—C16B	111.01 (12)
C13A—C14A—C15A	111.87 (13)	C13B—C14B—H14B	108.1
C13A—C14A—C16A	108.16 (13)	C15B—C14B—H14B	108.1
C15A—C14A—C16A	111.02 (15)	C16B—C14B—H14B	108.1
C13A—C14A—H13E	36.2	C14B—C15B—H15D	109.5
C15A—C14A—H13E	146.4	C14B—C15B—H15E	109.5
C16A—C14A—H13E	94.0	H15D—C15B—H15E	109.5
C13A—C14A—H14A	108.6	C14B—C15B—H15F	109.5
C15A—C14A—H14A	108.6	H15D—C15B—H15F	109.5
C16A—C14A—H14A	108.6	H15E—C15B—H15F	109.5
H13E—C14A—H14A	82.6	C14B—C16B—H16D	109.5
C15X—C14X—C16X	110.4 (11)	C14B—C16B—H16E	109.5
C15X—C14X—C13A	105.2 (9)	H16D—C16B—H16E	109.5
C16X—C14X—C13A	110.9 (10)	C14B—C16B—H16F	109.5
C15X—C14X—H14C	110.1	H16D—C16B—H16F	109.5
C16X—C14X—H14C	110.1	H16E—C16B—H16F	109.5
C13A—C14X—H14C	110.1	C5B—C17B—H17D	109.5
C14X—C15X—H15G	109.5	C5B—C17B—H17E	109.5
C14X—C15X—H15H	109.5	H17D—C17B—H17E	109.5
H15G—C15X—H15H	109.5	C5B—C17B—H17F	109.5
C14X—C15X—H15I	109.5	H17D—C17B—H17F	109.5
H15G—C15X—H15I	109.5	H17E—C17B—H17F	109.5
H15H—C15X—H15I	109.5	C19B—C18B—C23B	118.64 (10)
C14X—C16X—H16G	109.5	C19B—C18B—C3B	121.46 (10)
C14X—C16X—H16H	109.5	C23B—C18B—C3B	119.87 (10)
H16G—C16X—H16H	109.5	C20B—C19B—C18B	120.54 (11)
C14X—C16X—H16I	109.5	C20B—C19B—H19B	119.7
H16G—C16X—H16I	109.5	C18B—C19B—H19B	119.7
H16H—C16X—H16I	109.5	C21B—C20B—C19B	120.50 (10)
C5A—C17A—H17A	109.5	C21B—C20B—H20B	119.7
C5A—C17A—H17B	109.5	C19B—C20B—H20B	119.7
H17A—C17A—H17B	109.5	O6B—C21B—C20B	119.52 (10)
C5A—C17A—H17C	109.5	O6B—C21B—C22B	120.89 (10)
H17A—C17A—H17C	109.5	C20B—C21B—C22B	119.59 (10)
H17B—C17A—H17C	109.5	O5B—C22B—C23B	125.75 (10)
C19A—C18A—C23A	118.89 (10)	O5B—C22B—C21B	114.61 (10)
C19A—C18A—C3A	121.04 (10)	C23B—C22B—C21B	119.64 (11)
C23A—C18A—C3A	120.06 (10)	C22B—C23B—C18B	121.07 (10)
C18A—C19A—C20A	120.39 (11)	C22B—C23B—H23B	119.5
C18A—C19A—H19A	119.8	C18B—C23B—H23B	119.5
C20A—C19A—H19A	119.8	O5B—C24B—C25B	108.20 (10)
C21A—C20A—C19A	120.53 (10)	O5B—C24B—H24C	110.1
C21A—C20A—H20A	119.7	C25B—C24B—H24C	110.1
C19A—C20A—H20A	119.7	O5B—C24B—H24D	110.1
O6A—C21A—C20A	119.21 (10)	C25B—C24B—H24D	110.1
O6A—C21A—C22A	121.19 (10)	H24C—C24B—H24D	108.4
C20A—C21A—C22A	119.59 (10)	C24B—C25B—H25D	109.5
O5A—C22A—C23A	125.53 (10)	C24B—C25B—H25E	109.5
O5A—C22A—C21A	114.79 (10)	H25D—C25B—H25E	109.5
C23A—C22A—C21A	119.68 (10)	C24B—C25B—H25F	109.5
C22A—C23A—C18A	120.92 (10)	H25D—C25B—H25F	109.5
C22A—C23A—H23A	119.5	H25E—C25B—H25F	109.5
C18A—C23A—H23A	119.5		
			
C5A—N1A—C1A—C2A	15.77 (17)	C19A—C18A—C23A—C22A	−0.54 (18)
C5A—N1A—C1A—C6A	−162.96 (11)	C3A—C18A—C23A—C22A	178.13 (11)
N1A—C1A—C2A—C7A	−177.71 (10)	C22A—O5A—C24A—C25A	−177.04 (10)
C6A—C1A—C2A—C7A	0.82 (19)	C5B—N1B—C1B—C2B	−15.40 (17)
N1A—C1A—C2A—C3A	7.18 (16)	C5B—N1B—C1B—C6B	163.58 (11)
C6A—C1A—C2A—C3A	−174.29 (11)	N1B—C1B—C2B—C7B	176.69 (10)
C1A—C2A—C3A—C4A	−27.89 (14)	C6B—C1B—C2B—C7B	−2.12 (19)
C7A—C2A—C3A—C4A	156.54 (9)	N1B—C1B—C2B—C3B	−7.21 (16)
C1A—C2A—C3A—C18A	96.19 (12)	C6B—C1B—C2B—C3B	173.98 (11)
C7A—C2A—C3A—C18A	−79.37 (12)	C1B—C2B—C3B—C4B	27.93 (14)
C2A—C3A—C4A—C5A	29.86 (14)	C7B—C2B—C3B—C4B	−155.56 (9)
C18A—C3A—C4A—C5A	−94.63 (12)	C1B—C2B—C3B—C18B	−96.16 (13)
C2A—C3A—C4A—C12A	−152.16 (10)	C7B—C2B—C3B—C18B	80.35 (12)
C18A—C3A—C4A—C12A	83.34 (12)	C2B—C3B—C4B—C5B	−30.26 (14)
C12A—C4A—C5A—N1A	170.81 (10)	C18B—C3B—C4B—C5B	94.80 (13)
C3A—C4A—C5A—N1A	−11.24 (16)	C2B—C3B—C4B—C12B	153.23 (10)
C12A—C4A—C5A—C17A	−9.67 (18)	C18B—C3B—C4B—C12B	−81.70 (12)
C3A—C4A—C5A—C17A	168.28 (11)	C12B—C4B—C5B—N1B	−171.58 (10)
C1A—N1A—C5A—C4A	−13.72 (17)	C3B—C4B—C5B—N1B	11.96 (16)
C1A—N1A—C5A—C17A	166.70 (10)	C12B—C4B—C5B—C17B	9.25 (18)
C8A—O1A—C7A—O2A	7.54 (16)	C3B—C4B—C5B—C17B	−167.21 (11)
C8A—O1A—C7A—C2A	−171.77 (10)	C1B—N1B—C5B—C4B	12.98 (17)
C1A—C2A—C7A—O2A	−176.47 (11)	C1B—N1B—C5B—C17B	−167.74 (10)
C3A—C2A—C7A—O2A	−1.10 (16)	C8B—O1B—C7B—O2B	−3.28 (16)
C1A—C2A—C7A—O1A	2.84 (16)	C8B—O1B—C7B—C2B	176.86 (10)
C3A—C2A—C7A—O1A	178.21 (9)	C1B—C2B—C7B—O2B	178.67 (11)
C7A—O1A—C8A—C9A	−169.38 (10)	C3B—C2B—C7B—O2B	2.34 (16)
O1A—C8A—C9A—C11A	68.38 (14)	C1B—C2B—C7B—O1B	−1.47 (17)
O1A—C8A—C9A—C10A	−57.88 (14)	C3B—C2B—C7B—O1B	−177.79 (9)
C13A—O3A—C12A—O4A	−3.12 (18)	C7B—O1B—C8B—C9B	173.07 (10)
C13A—O3A—C12A—C4A	175.20 (11)	O1B—C8B—C9B—C11B	59.58 (14)
C5A—C4A—C12A—O4A	−9.69 (19)	O1B—C8B—C9B—C10B	−177.11 (11)
C3A—C4A—C12A—O4A	172.35 (12)	C13B—O3B—C12B—O4B	0.82 (17)
C5A—C4A—C12A—O3A	172.09 (10)	C13B—O3B—C12B—C4B	−177.78 (10)
C3A—C4A—C12A—O3A	−5.88 (15)	C5B—C4B—C12B—O4B	12.61 (19)
C12A—O3A—C13A—C14A	179.13 (12)	C3B—C4B—C12B—O4B	−170.91 (12)
C12A—O3A—C13A—C14X	−139.8 (5)	C5B—C4B—C12B—O3B	−168.86 (10)
O3A—C13A—C14A—C15A	60.00 (18)	C3B—C4B—C12B—O3B	7.62 (15)
C14X—C13A—C14A—C15A	−40.6 (7)	C12B—O3B—C13B—C14B	−178.14 (11)
O3A—C13A—C14A—C16A	−177.42 (15)	O3B—C13B—C14B—C15B	−64.79 (16)
C14X—C13A—C14A—C16A	82.0 (7)	O3B—C13B—C14B—C16B	171.70 (12)
O3A—C13A—C14X—C15X	−62.3 (9)	C4B—C3B—C18B—C19B	−62.41 (14)
C14A—C13A—C14X—C15X	31.6 (7)	C2B—C3B—C18B—C19B	61.03 (14)
O3A—C13A—C14X—C16X	178.3 (10)	C4B—C3B—C18B—C23B	115.73 (12)
C14A—C13A—C14X—C16X	−87.8 (12)	C2B—C3B—C18B—C23B	−120.84 (11)
C4A—C3A—C18A—C19A	59.76 (14)	C23B—C18B—C19B—C20B	−0.07 (17)
C2A—C3A—C18A—C19A	−63.48 (14)	C3B—C18B—C19B—C20B	178.08 (11)
C4A—C3A—C18A—C23A	−118.89 (12)	C18B—C19B—C20B—C21B	−0.09 (18)
C2A—C3A—C18A—C23A	117.87 (12)	C19B—C20B—C21B—O6B	179.83 (11)
C23A—C18A—C19A—C20A	0.14 (18)	C19B—C20B—C21B—C22B	−0.50 (18)
C3A—C18A—C19A—C20A	−178.51 (11)	C24B—O5B—C22B—C23B	8.62 (17)
C18A—C19A—C20A—C21A	−0.07 (18)	C24B—O5B—C22B—C21B	−171.60 (10)
C19A—C20A—C21A—O6A	179.50 (11)	O6B—C21B—C22B—O5B	1.11 (16)
C19A—C20A—C21A—C22A	0.37 (18)	C20B—C21B—C22B—O5B	−178.55 (10)
C24A—O5A—C22A—C23A	−8.52 (17)	O6B—C21B—C22B—C23B	−179.09 (11)
C24A—O5A—C22A—C21A	172.11 (10)	C20B—C21B—C22B—C23B	1.24 (17)
O6A—C21A—C22A—O5A	−0.46 (16)	O5B—C22B—C23B—C18B	178.35 (11)
C20A—C21A—C22A—O5A	178.65 (11)	C21B—C22B—C23B—C18B	−1.42 (18)
O6A—C21A—C22A—C23A	−179.87 (11)	C19B—C18B—C23B—C22B	0.83 (17)
C20A—C21A—C22A—C23A	−0.76 (17)	C3B—C18B—C23B—C22B	−177.35 (10)
O5A—C22A—C23A—C18A	−178.49 (11)	C22B—O5B—C24B—C25B	177.15 (10)
C21A—C22A—C23A—C18A	0.85 (18)		

### Hydrogen-bond geometry (Å, °)

**Table:**

Cg2 and Cg4 are the centroids of the C18*A*–C23*A* and C18*B*–C23*B* rings, respectively.

**Table:**

*D*—H···*A*	*D*—H	H···*A*	*D*···*A*	*D*—H···*A*
N1B—H1NB···O6A^i^	0.896 (16)	2.077 (16)	2.9432 (14)	162.3 (15)
N1A—H1NA···O6B^ii^	0.893 (18)	2.067 (18)	2.9360 (14)	164.1 (16)
O6B—H1OB···O2B^ii^	0.83 (2)	1.98 (2)	2.7339 (13)	149.9 (19)
O6A—H1OA···O2A^iii^	0.83 (2)	1.94 (2)	2.6992 (13)	153 (2)
C11A—H11C···O4B^iv^	0.98	2.52	3.4755 (19)	164
C16A—H16A···O4A^v^	0.98	2.59	3.316 (2)	131
C24A—H24B···Cg2^iii^	0.99	2.99	3.7291 (15)	132
C24B—H24C···Cg4^ii^	0.99	2.94	3.6754 (14)	132
C14X—H14C···Cg4^vi^	1.00	2.98	3.978 (12)	172

Symmetry codes: (i) −*x*, −*y*+1, −*z*+1; (ii) −*x*, −*y*+1, −*z*; (iii) −*x*+1, −*y*+1, −*z*+1; (iv) *x*+1, *y*−1, *z*; (v) −*x*+1, −*y*+2, −*z*; (vi) *x*+1, *y*, *z*.
